# Effects of *Bacillus megatherium* 1259 on Growth Performance, Nutrient Digestibility, Rumen Fermentation, and Blood Biochemical Parameters in Holstein Bull Calves

**DOI:** 10.3390/ani11082379

**Published:** 2021-08-12

**Authors:** Bobo Deng, Yinyin Chen, Xiaoxiao Gong, Yi Dai, Kang Zhan, Miao Lin, Lin Wang, Guoqi Zhao

**Affiliations:** 1 College of Animal Science and Technology, Yangzhou University, Yangzhou 225009, China; 18168960899@163.com (B.D.); chenyinyin90s@163.com (Y.C.); 18762329160@163.com (X.G.); cminghui93@gmail.com (Y.D.); zhankang0305@163.com (K.Z.); linmiao3025@163.com (M.L.); 2 Institutes of Agricultural Science and Technology Development, Yangzhou University, Yangzhou 225009, China; michelle861215@hotmail.com; 3 Joint International Research Laboratory of Agriculture and Agri-Product Safety, The Ministry of Education of China, Yangzhou University, Yangzhou 225009, China

**Keywords:** *Bacillus megaterium* 1259, growth performance, nutrient digestibility, rumen fermentation, blood biochemical parameters, Holstein bull calves

## Abstract

**Simple Summary:**

This study was conducted to investigate the effects of dietary supplementation with *Bacillus megaterium* 1259 (BM1259) on growth performance, nutrient digestibility, rumen fermentation, and blood biochemical parameters in Holstein bull calves. The results demonstrated that the addition of BM1259 to the diets can significantly improve the growth performance and elevate the apparent digestibility of crude protein and neutral detergent fiber. Moreover, supplementation with BM1259 ameliorated rumen fermentation and reduced the emission of both ammoniacal nitrogen and sulfuretted hydrogen in feces and urine. In addition, adding 12 g/head/day of BM1259 had no adverse effect on blood biochemical parameters and the health status of Holstein bull calves. This study demonstrates that BM1259 can be applied as a potential microecologics to improve production performance and nitrogen utilization in Holstein bull calves.

**Abstract:**

*Bacillus megaterium* is an ideal microecologics in the feed industry. BM1259 was already isolated from chicken manure and the whole-genome sequencing was also analyzed in our previous study. However, few studies concentrated on dietary supplementation with BM1259 in young ruminants and especially its effect on Holstein bull calves have not been reported. Hence, this experiment was conducted with the aim to evaluate the effects of BM1259 on growth performance, nutrient digestibility, rumen fermentation, and blood biochemical parameters in Holstein bull calves. Twenty-four healthy Holstein bull calves with the initial age of 90 days old and a similar body weight (115 ± 6.5 kg) were selected and randomly allocated into two groups with one Holstein bull calf in each pen (2.5 m × 2.2 m). Holstein bull calves in the control group (COG) were fed a basal total mixed ration (TMR), while experimental treatments (BMG) were fed with the TMR diet supplemented with 12 g/head/day of BM1259 powder (1 × 10^10^ cfu/g) separately. Results showed that (1) the average daily gain and dry matter intake of the BMG were significantly higher than those of the COG (*p* < 0.01), increased by 12.5% and 8.79%, respectively, during the 4–8 weeks after the addition of 12 g/head/day of BM1259; from 0 to 8 weeks, ADG (*p* < 0.05) and DMI (*p* < 0.05) in the BMG were significantly higher than those in the COG, increased by 14.9% and 6.04%, respectively. (2) At the end of the fourth week, the apparent digestibility of crude protein and neutral detergent fiber in the BMG was significantly higher than that in the COG (*p* < 0.05), increased by 5.97% and 6.70%, respectively; at the end of the eighth week, the apparent digestibility of crude protein and neutral detergent fiber was significantly higher than that of the COG (*p* < 0.01), increased by 5.88% and 10.26%, respectively. (3) At the end of the eighth week, the rumen fluid pH (*p* < 0.05), MCP (*p* < 0.05), and acetate (*p* < 0.05) in the BMG were significantly higher than those in the COG, increased by 9.03%, 19.68%, and 12.74%, respectively; at the end of the fourth and eighth week, NH_3_-N concentration in the BMG was significantly lower than that in the COG, with a decrease of 21.81% and 16.40%, respectively. (4) At the end of the fourth (*p* < 0.05) and eighth week (*p* < 0.05), the glutamate content of the rumen fluid of the Holstein bull calves in the BMG was significantly higher than that in the COG, increased by 13.21% and 14.32%, respectively; at the end of the fourth week, the contents of glutamate in the serum (*p* < 0.05), urine (*p* < 0.05), and feces (*p* < 0.05) of the Holstein bull calves in the BMG were significantly lower than those in the COG, decreased by 25.76%, 33.87%, and 9.23%, respectively; at the end of the eighth week, the contents of glutamate in the serum, urine, and feces of the Holstein bull calves in the BMG were significantly lower than those in the COG (*p* < 0.01), decreased by 26.69%, 27.94%, and 11.11%, respectively. (5) After adding 12 g/head/day of BM1259, the urine ammonia–nitrogen content of the BMG was extremely significantly lower than that of the COG at the end of the fourth and eighth week (*p* < 0.01), decreased by 54.60% and 40.31%, respectively. (6) After adding 12 g/head/day of BM1259, there was no significant effect on the level of blood biochemical parameters of the Holstein bull calves. This study demonstrates that BM1259 can be applied as a potential microecologics to improve growth performance, nutrient digestibility, rumen fermentation, and nitrogen utilization in Holstein bull calves.

## 1. Introduction

In recent years, research and application of microecologics has attracted extensive attention from scholars in domestic fields and abroad. As a new kind of green and environmental protection feed additive, microecologics have been widely used in animal husbandry due to its green, safe, and residue-free function, which can be used to replace antibiotics, to promote the healthy growth of animals, and improve the environment [1,2]. At present, lactic acid bacteria, bacillus, yeast, photosynthetic bacteria, and so on are the most widely studied microecological additives. Among them, bacillus has a history of more than 100 years since its discovery and a large quantity of scientific research data have confirmed the use effect of bacillus [3]. Among bacillus, *Bacillus megatherium* as probiotics has become a research hotspot due to its unique characteristics, such as its strong resistance to stress, high temperature resistance, and easy storage [4,5]. BM1259, as a new-type of microecological additive isolated from chicken manure, is a gram-positive bacterium that can produce spores. BM1259 is usually a rode-shaped bacterium with rounded ends and tends to be arranged in single or chain forms. It is a large bacterium with a diameter of more than 1.0 μm and a volume of more than 60 microcubic meters, which is 100 times the volume of E. coli, demonstrating strong resistance to high temperature treatment, ultraviolet radiation, drying, and high concentrations of organic solvents. It plays a very important role in improving animal production performance and product quality, increasing the output of livestock products and reducing feed costs [6,7,8,9,10]. Kritas et al. demonstrated that supplementing ewe feed with bacillus sp. probiotics promoted subsequent milk with yields, fat, and protein contents [11]. Huo et al. showed that feeding 0.04% of BM1259 could improve the digestibility of crude protein and dry matter in the diet of finishing pigs, reduce the production of NH_3_, and improve the air environment of pig pens to a certain extent [12]. Ding et al. added 100 mg/kg BM1259 to the feed of laying hens and found that the parameters of the total egg weight, egg number, and average egg weight were significantly increased, while the contents of ammonia–nitrogen, uric acid, and urea nitrogen in the excrement were significantly decreased, which could improve economic benefits [13]. Yu et al. supplemented 12 g/head/day of BM1259 to the diet of dairy cows, finding that the concentrations of urea nitrogen and uric acid in feces decreased, the activities of urease and uricase decreased, and the activity of alanine aminotransferase in the serum also decreased [14].

However, few studies concentrated on dietary supplementation with BM1259 in young ruminants and especially the study and application of BM1259 on Holstein bull calves is still unknown. Hence, this work was conducted with the aim to evaluate the effects of BM1259 on growth performance, nutrient digestibility, rumen fermentation, and blood biochemical parameters in Holstein bull calves. Together, these data could represent a useful basis for future applications of BM1259 as ruminant microecologics.

## 2. Materials and Methods

### 2.1. Ethical Considerations

All Holstein bull calves used in this study were strictly cared in accordance with the principles of Yangzhou University, the Institutional Animal Care, and the Use Committee (SYXK (Su) IACUC 2016-0019).

### 2.2. Experimental Design, Animals, Diets, and Feeding Management

The experiment was conducted at the Ruminant Experiment Research Farm, Yangzhou University (China), from April to July 2020. Twenty-four healthy Holstein bull calves of 90 days old with similar body weights (115 ± 6.5 kg) were selected and randomly allocated into two groups with 12 replicates in each group and 1 Holstein bull calf in each pen (2.5 m × 2.2 m). Holstein bull calves in the control group were fed a basal total mixed ration (TMR) without BM1259 and the BMG were fed with the TMR supplemented with 12 g/head/day (recommended dose for the preliminary experiment) BM1259 powder (1 × 10^10^ cfu/g) separately. TMR were adjusted as isonitrogenous and isoenergetic diets (Table 1). The basal diets were formulated to meet or exceed the nutritional requirements and feeding standards of NRC (2001) cows. During the whole experiment period, all Holstein bull calves were given ad libitum access to TMR and fresh tap water. The pen was disinfected before the experiment began. Holstein bull calves were fed twice at 8:00 am and 17:00 pm each day. Daily feed allocations to each pen were adjusted according to the minimal feed refusals (<10%) in the feed bunk and the weight of the residual was recorded every day before new feeding was delivered. This experiment consisted of a 14-day adaptation and a 56-day fattening period for sample collection.

### 2.3. Sample Collection and Parameter Measurement

The offered and refused feed amounts were recorded on the third and fourth days every other week throughout the entire experimental period. All samples were dried in an oven at 65 °C for 48 h, ground with a Wiley mill to pass through a 2-mm screen, and then stored in a refrigerator at −20 °C for further analyses. The daily feed offered, orts, and spillages were collected and weighed to determine the average daily feed intake. At the end of 4th week and 8th week, withers height, body length, and chest girth were measured as quantitative indicators.

At the 7th day, the digestion and metabolism test was conducted at the end of the formal period, in which the first two days were the adaptation period and the last five days were sampled. Fecal samples were collected 3 times a day with an interval of 8 h for each time. After the collection, 10% H_2_SO_4_ was added for nitrogen fixation. The 5-day fecal samples (200 g) were mixed and stored in a refrigerator at −20 °C for the determination of the apparent digestibility of nutrients. Feed and feces were dried and ground using a pulverizer for the subsequent determination of dry matter, crude protein, neutral detergent fiber, acid detergent fiber, ether extract, and crude ash content. For both TMR and fecal samples, DM was detected using AOAC method 930.15; EE was measured using AOAC method 920.85; CP was tested using the method described by Kjeldahl with an azotometer (Scino KT260, FOSS, Hillerod, Denmark); NDF and ADF were measured using the methods described by Van Soest and a fiber analyzer (2000i, Ankom, New York, NY, USA); and ash content was measured according to AOAC method 938.08 [15].

On the last day of the experiment, the rumen fluid (250 mL) was collected through an oral collector and filtered with a four-layer sterilized gauze two hours after the morning feeding. The pH (PB-21, Beijing Sartorius Scientific Instrument Co., Ltd., Bejing, China) value was determined immediately. Then, 10 mL aliquots were added with 0.1 mL of 6 mol/L HCl and stored at −20 °C for the determination of the ammonia–nitrogen concentration according to the method of Weatherburn [16]. Another 10 mL of ruminal fluid samples were thawed in ultrapure water and centrifuged for 10 min at 15,000× *g*, after which supernatants were removed to centrifuge tubes containing 25% metaphosphoric acid and then stored at −20 °C for the analysis of volatile fatty acid concentration using a GC Ultra gas chromatograph (Thermo Fisher Scientific, Waltham, MA, USA) as described by Guo et al. [17]. The yield of crude microbial proteins was determined according to the method described by Hall and Herejk [18].

Jugular blood samples of approximately 10 mL were collected from each Holstein bull calf in vacuum plasma tubes at 3 h after the morning feeding on the third day of weeks 4 and 8 [19]. The collected blood was then centrifuged at 3000× *g* at 4 °C for 10 min to collect serum, which was then frozen at −20 °C for further analysis. Serum samples were analyzed for the quantification of the total protein (TP), albumin (ALB), globulin (GLOB), aspartate aminotransferase (AST), alanine aminotransferase (ALT), blood urea nitrogen (BUN), and uric acid (UA) according to methods described in previous studies [20].

The ITC pre-column derivatization method was used to determine glutamate. The specific method was in reference to the research report of J.L. Zhang et al. [21]. The concentration of NH_3_-N in the urine was determined by method of phenol–sodium hypochlorite colorimetry [22].

### 2.4. Statistical Analysis

The numerical data were analyzed by one-way analysis of variance (ANOVA) with orthogonal data and the significance of differences between the groups was determined by Tukey’s post-hoc test, assuming significance levels of 0.05 and 0.01. The tables present the means and standard error of the mean (SEM). The calculations were made using SAS 9.4 software (SAS Institute, Cary, NC, USA).

## 3. Results

### 3.1. Influence of BM1259 on the Growth Performance and Body Size of Holstein Bull Calves

As shown in Table 2, the ADG and DMI of the BMG were significantly higher than those of the control group (*p* < 0.01), increased by 12.5% and 8.79%, respectively, during the 4–8 weeks after the addition of 12 g/head/day of BM1259; from 0 to 8 weeks, ADG (*p* < 0.05) and DMI (*p* < 0.05) in the BMG were significantly higher than those in the control group, increased by 14.9% and 6.04%, respectively. The feed conversion rate, withers height, body length, and chest girth were higher in the BMG but the differences were not significant (*p* > 0.05).

### 3.2. Effects of BM1259 on the Nutrient Apparent Digestibility of Holstein Bull Calves

As is presented in Table 3, at the end of the fourth week, the apparent digestibility of CP and NDF in the BMG was significantly higher than that in the COG (*p* < 0.05), increased by 5.97% and 6.70%, respectively, after adding 12 g/head/day of BM1259. At the end of the eight week, the apparent digestibility of CP and NDF was significantly higher than that of the control group (*p* < 0.01), increased by 5.88% and 10.26%, respectively. Compared with the COG, the apparent digestibility of ADF and EE in the BMG was higher but there was no significant difference (*p* > 0.05).

### 3.3. Influence of BM1259 on the Rumen Fermentation Parameters

As is shown in Table 4, at the end of the eight week, the rumen fluid pH (*p* < 0.05), microbial protein (*p* < 0.05), and acetate (*p* < 0.05) in the BMG were significantly higher than those in the COG, increased by 9.03%, 19.68%, and 12.74%, respectively, after adding 12 g/head/day of BM1259. At the end of the fourth and eighth week, NH_3_-N concentration in the BMG was significantly lower than that in the COG, with a decrease of 21.81% and 16.40%, respectively. The content of sulfuretted hydrogen, total volatile fatty acids, propionate, butyrate, and acetate:propionate showed no significant differences between the COG and BMG at each stage of experiment (*p* > 0.05).

### 3.4. Effect of BM1259 on the Contents of Glutamate in the Rumen Fluid, Serum, Feces, and Urine

In this study, in order to explore the effects of adding 12 g/head/day of BM1259 on the glutamate content, the glutamate content was measured in the rumen fluid, serum, feces, and urine of the Holstein bull calves in the BMG and COG (Table 5). Results demonstrated that at the end of the fourth (*p* < 0.05) and eighth week (*p* < 0.05), the glutamate content of the rumen fluid in the BMG was significantly higher than that in the COG, increased by 13.21% and 14.32%, respectively; at the end of the fourth week, the contents of glutamate in the serum (*p* < 0.05), urine (*p* < 0.05), and feces (*p* < 0.05) of the Holstein bull calves in the BMG were significantly lower than those in the COG, decreased by 25.76%, 33.87%, and 9.23%, respectively; at the end of the eighth week, the contents of glutamate in the serum, urine, and feces in the BMG were significantly lower than those in the COG (*p* < 0.01), decreased by 26.69%, 27.94%, and 11.11%, respectively.

### 3.5. Effects of BM1259 on the Content of H_2_S and Ammonia–Nitrogen in Urine

As is shown in Table 6, by adding 12 g/head/day of BM1259, the urine ammonia–nitrogen content of the BMG was extremely significantly lower than that of the COG at the end of the fourth and eighth week (*p* < 0.01), decreased by 54.60% and 40.31%, respectively. H_2_S content in the urine of the BMG decreased overall but there was no significant difference between the two groups (*p* > 0.05).

### 3.6. Effect of BM1259 on the Blood Biochemical Parameters of Holstein Bull Calves

As is presented in Table 7, compared with the COG, adding 12 g/head/day of BM1259 had no significant effect on the level of blood biochemical parameters of Holstein bull calves.

## 4. Discussion

It was found that adding bacillus to the diet of Holstein bull calves could improve the performance of Holstein bull calves. Youssef et al. showed that feeding bacillus subtilis BSN fermentation significantly improved the feed conversion rate and milk yield but had no significant effects on the milk fat percentage and milk protein percentage [23]. Hu et al. demonstrated that feeding 1.0 × 10^10^ cfu/g bacillus subtilis improved the milk yield of Holstein cows by 11.61% [24]. Deng et al. found that feeding bacillus natto significantly increased the milk yield of 4% milk fat [25]. It was found that the addition of 12 g/head/day of BM1259 could significantly improve the growth performance and rumen fermentation ability, and there was no significant difference in the blood biochemical parameters. In our study, the ADG and DMI were significantly increased but there were no significant differences in FCR, WH, and BL. During the growth and metabolism of bacillus, some digestive enzymes with strong activity can be produced, such as protease, lipase, and starch hydrolase, which can decompose some complex carbohydrates such as gum and xylan, so as to promote the digestion and absorption capacity of nutrients in the rumen [26]. In this study, we found that supplementation of 12 g/head/day of BM1259 could significantly improve the digestion and utilization ratio of CP and glutamate, which was related to 12 coding genes related to nitrogen metabolism in our previous research. In addition, the digestion and utilization rate of NDF were significantly improved after the addition of 12 g/head/day of BM1259, which might be due to the secretion of certain digestive enzymes that promoted the decomposition and digestion of NDF.

The rumen pH value is one of the evaluation parameters of rumen microbial growth, metabolism balance, and fermentation degree, which is mainly affected by the amount of saliva buffer secretion of ruminants [27]. The rumen fibro lytic bacteria had the highest activity at pH 6.7 and the rumen degradability and acetate content decreased with the decrease of pH [28,29,30]. In this study, after adding 12 g/head/day of BM1259, the rumen pH value increased significantly from 6.20 to 6.76 after eight weeks. The results indicated that adding a certain amount of BM1259 could promote the growth of fibro lytic bacteria. NH_3_-N is the main nitrogen source (about 20–100%) in the process of rumen microbial flora growth and reproduction of ruminants. The concentration of NH_3_-N should be moderate in order to facilitate microbial growth and promote microbial MCP generation. Too high or too low concentration is not suitable for microbial growth [31,32,33]. In this study, it was found that the rumen NH_3_-N concentration was significantly decreased after the addition of 12 g/head/day of BM1259, which was in the range of 8.46–12.03 mg/dL, although lower than that of the 10 g/head/day of BM1259 (15.95–25.32 mg/dL) in Yu [34]. However, dietary supplementation of BM1259 has a certain influence on the rumen ammonia–nitrogen NH_3_-N concentration. VFA is one of the energy substances used to maintain the normal life activities of ruminants, which can provide more than 70% of the digestive energy and participate in different metabolisms in the body [35]. Diana et al. found that by feeding bacillus subtilis, C2 concentration and C2:C3 were significantly decreased, while TVFA, C3, and NH_3_-N contents were significantly increased [36]. Ding et al. found that the C2:C3 could be significantly reduced and both the TVFA and C3 content could be increased with supplementation of bacillus subtilis in the fermentation test [37]. However, our results demonstrated that after the addition of BM1259, the C2 concentration increased significantly and the C2:C3 increased, indicating that the rumen fermentation type changed to acetate fermentation, which was consistent with the significant increase in the content of *Saccharofermentans* in our previous study [2]. The levels of NH_3_-N and H_2_S in the urine not only affected the health of the livestock and humans, but also reflected the nitrogen utilization capacity of animals [38]. In this study, after the addition of 12 g/head/day of BM1259, NH_3_-N in the urine of Holstein bull calves significantly decreased and the content of H_2_S in the excreta was significantly reduced.

The level of the blood biochemical parameters can reflect the status of nutrient metabolism and the acid-base balance of the animal body to a certain extent. Meanwhile, the level of serum albumin and total protein is an indicator for measuring the health status of the animal [39,40,41]. In this study, it was found that after adding 12 g/head/day of BM1259, most biochemical parameters in the blood of Holstein bull calves did not significantly change, indicating that BM1259 did not have adverse effects on the body health status.

## 5. Conclusions

In summary, the addition of BM1259 to the diets of Holstein bull calves can significantly improve the growth performance and elevate the apparent digestibility of CP and NDF. Moreover, supplementation with BM1259 increased C2 and C2:C3 in the rumen, and reduced emission of NH_3_-N and H_2_S in the feces and urine, indicating a possible amelioration in the rumen fermentation and nitrogen utilization. In addition, adding 12 g/head/day of BM1259 had no adverse effect on blood biochemical parameters and health status. However, further research is necessary to uncover the mechanism by which BM1259 improves nitrogen utilization in Holstein bull calves.

## Figures and Tables

**Table 1:** Basal diet formulations and nutritional contents (DM basis %).

Items	Content	Nutrient Levels ^(2)^	Content
Ground corn	23.79	ME (MJ/kg)	11.93
Wheat bran	10.49	DM (%)	89.18
Spray corn husk	3.49	CP (%DM)	18.85
Soybean meal (46%)	10.35	EE (%DM)	3.22
DDGS (corn)	7.00	NDF (%DM)	41.08
Corn germ meal	7.00	ADF (%DM)	19.13
Soybean hull	3.50	Calcium (%DM)	0.85
Salt	0.35	Phosphorus (%DM)	0.41
Calcium carbonate	1.23	Glu (%DM)	1.69
Oat hay	30.00		
Premix ^(1)^	2.80		
Total	100.00		

Abbreviations: DDGS, distiller dried grains with soluble; ME, metabolic energy; DM, dry matter; CP, crude protein; EE, ether extract; NDF, neutral detergent fiber; ADF, acid detergent fiber; and Glu, glutamate. ^(1)^ Premix was formulated to provide (per kilogram of the dietary DM) 60.5 mg of Zn as ZnSO4·7H_2_O; 58.5 mg of Mn as MnSO_4_·H_2_O; 72.5 mg of Fe as FeSO_4_·7H_2_O; 30.75 mg of Cu as CuSO_4_·5H_2_O; 0.85 mg of I as KI; 0.45 mg of Co as CoCl_2_·6H_2_O; 0.63 mg of Se as Na_2_Se·NO_3_; 3150 ten thousand IU of vitamin A; 3500 IU of vitamin D; 20 ten thousand mg of vitamin E; and 4200 mg of vitamin K_3_. ^(2)^ Metabolic energy was a calculated value, while the others were measured values; ME = (36.21 × CP + 85.44 × EE + 37.26 × NFE) × 4.184.

**Table 2:** Influence of BM1259 on growth performance and body size of Holstein bull calves.

Item	Time	COG	BMG	SEM	*p*-Value
ADG (kg/d)	0–4 week	1.24	1.37	0.02	*p* < 0.05
	4–8 week	1.52	1.71	0.03	*p* < 0.01
	0–8 week	1.34	1.54	002	*p* < 0.05
DMI (kg/d)	0–4 week	4.58	4.68	0.04	0.23
	4–8 week	6.03	6.56	0.05	*p* < 0.01
	0–8 week	5.30	5.62	0.03	*p* < 0.05
FCR	0–4 week	3.75	3.44	0.10	0.05
	4–8 week	4.00	3.88	0.11	0.46
	0–8 week	4.02	3.67	0.12	0.06
WH (cm)	4th weekend	93.10	94.60	0.75	0.89
	8th weekend	136.90	142.30	2.19	0.37
BL (cm)	4th weekend	91.50	106.10	1.15	0.39
	8th weekend	147.70	158.50	1.84	0.73
CG (cm)	4th weekend	161.50	173.80	2.50	0.83
	8th weekend	175.00	199.60	2.15	0.32

Abbreviations: COG, control group; BMG, *bacillus megatherium* group; SEM, standard error of the mean; ADG, average daily gain; DMI, dry matter intake; FCR, feed conversion rate; WH, withers height; BL, body length; and CG, chest girth.

**Table 3:** Effects of BM1259 on the nutrient apparent digestibility of Holstein bull calves.

Item	Time	COG	BMG	SEM	*p*-Value
CP (%)	4th weekend	59.93	63.51	0.86	*p* < 0.05
	8th weekend	61.40	65.01	0.45	*p* < 0.01
NDF (%)	4th weekend	53.70	57.30	0.93	*p* < 0.05
	8th weekend	55.67	61.38	0.85	*p* < 0.01
ADF (%)	4th weekend	45.56	48.36	0.83	0.05
	8th weekend	49.94	51.65	0.80	0.07
EE (%)	4th weekend	71.33	72.48	2.32	0.74
	8th weekend	73.03	75.65	1.68	0.30

Abbreviations: CP, crude protein; NDF, neutral detergent fiber; ADF, acid detergent fiber; and EE, ether extract.

**Table 4:** Influence of BM1259 on the rumen fermentation parameters of Holstein bull calves.

Item	Time	COG	BMG	SEM	*p*-Value
PH	4th weekend	6.33	6.45	0.07	0.35
	8th weekend	6.20	6.76	0.05	*p* < 0.05
NH_3_-N (mg/dL)	4th weekend	10.82	8.46	0.18	*p* < 0.01
	8th weekend	14.39	12.03	0.82	*p* < 0.01
MCP (mg/mL)	4th weekend	2.23	2.63	0.05	*p* < 0.05
	8th weekend	2.49	2.98	0.05	*p* < 0.05
H_2_S (nmol/mL)	4th weekend	14.11	12.55	1.09	0.32
	8th weekend	13.5	12.11	0.69	0.16
TVFA (mmol/L)	4th weekend	95.88	113.29	6.85	0.06
	8th weekend	99.60	107.34	3.41	0.45
Acetate (C2)(mmol/L)	4th weekend	53.69	66.83	3.44	*p* < 0.05
	8th weekend	56.69	63.91	2.44	*p* < 0.05
Propionate(C3) (mmol/L)	4th weekend	24.18	28.74	1.94	0.12
	8th weekend	24.94	27.07	1.46	0.31
Butyrate(C4) (mmol/L)	4th weekend	12.19	15.77	1.21	0.06
	8th weekend	12.18	13.76	0.89	0.24
C2:C3	4th weekend	2.27	2.43	0.16	0.48
	8th weekend	2.36	2.46	0.21	0.36

Abbreviation: MCP, microbial protein; TVFA, total volatile fatty acids; C2, acetate; C3, propionate; and C4, butyrate.

**Table 5:** Effect of BM1259 on the contents of glutamate in the rumen fluid, serum, feces, and urine of Holstein bull calves.

Item	Time	COG	BMG	SEM	*p*-Value
Rumen fluid (g/100 mL)	4 weekend	3.86	4.37	0.15	*p* < 0.05
	8 weekend	4.19	4.79	0.17	*p* < 0.05
Serum (g/100 mL)	4 weekend	3.30	2.45	0.11	*p* < 0.05
	8 weekend	3.41	2.50	0.09	*p* < 0.01
Urine (g/100 mL)	4 weekend	0.62	0.41	0.03	*p* < 0.05
	8 weekend	0.68	0.49	0.03	*p* < 0.01
Feces (mg/g DM)	4 weekend	0.65	0.59	0.02	*p* < 0.05
	8 weekend	0.72	0.64	0.03	*p* < 0.01

**Table 6:** Effects of BM1259 on the content of H_2_S and ammonia–nitrogen in urine.

Item	Time	COG	BMG	SEM	*p*-Value
NH_3_-N (mg/dL)	4th weekend	1.74	0.79	0.16	*p* < 0.01
	8th weekend	1.91	1.14	0.13	*p* < 0.01
H_2_S (nmol/mL)	4th weekend	7.79	6.78	1.39	0.36
	8th weekend	7.36	7.12	0.81	0.21

**Table 7:** Effect of BM1259 on the blood biochemical parameters of Holstein bull calves.

Item	Time	COG	BMG	SEM	*p*-Value
ALT(U/L)	4th weekend	28.76	21.92	3.81	0.14
	8th weekend	24.76	25.84	3.74	0.48
AST(U/L)	4th weekend	85.84	85.61	8.82	0.05
	8th weekend	84.15	94.46	8.58	0.09
TP (g/L)	4th weekend	63.68	61.99	7.40	0.57
	8th weekend	64.56	64.72	4.87	0.94
ALB (g/L)	4th weekend	28.12	27.86	1.38	0.64
	8th weekend	29.12	29.72	0.69	0.32
GLO (g/L)	4th weekend	35.56	34.13	6.70	0.60
	8th weekend	35.44	35.00	4.19	0.79
BUN (mmoI/L)	4th weekend	5.38	5.49	1.40	0.85
	8th weekend	3.44	3.94	1.20	0.30
UA (mmoI/L)	4th weekend	27.84	28.30	2.70	0.96
	8th weekend	30.61	28.84	2.36	0.38

Abbreviations: ALT, alanine transaminase; AST, aspartate transaminase; TP, total protein; ALB, albumin; GLO, globulin; BUN, blood urea nitrogen; and UA, uric acid.

## Data Availability

Not applicable.
